# Combining Geriatric Nutritional Risk Index with Total Cholesterol to Predict Pneumonia Mortality Risks in a Cohort of General Older Adults

**DOI:** 10.3390/nu18030465

**Published:** 2026-01-30

**Authors:** Rui Yu, Tatsuma Okazaki, Yilin Du, Naoki Suzuki, Takahiro Miura, Midori Miyagi, Mana Kogure, Naoki Nakaya, Atsushi Hozawa, Satoru Ebihara

**Affiliations:** 1 Department of Rehabilitation Medicine, Tohoku University Graduate School of Medicine, 1-1 Seiryo-machi, Aoba-Ku, Sendai 980-8574, Japan; yu.rui.p4@dc.tohoku.ac.jp (R.Y.); naoki.suzuki.e3@tohoku.ac.jp (N.S.);; 2 Department of Preventive Medicine and Epidemiology, Tohoku Medical Megabank Organization, 2-1 Seiryo-machi, Aoba-Ku, Sendai 980-8573, Japannaoki.nakaya.c2@tohoku.ac.jp (N.N.); 3 Division of Epidemiology, Graduate School of Medicine, Tohoku University, 1-1 Seiryo-machi, Aoba-Ku, Sendai 980-8574, Japan

**Keywords:** geriatric nutritional risk index, total cholesterol, pneumonia mortality, older people, TC-GNRI

## Abstract

**Background/Objectives:** To examine the importance of a composite measure incorporating the Geriatric Nutritional Risk Index (GNRI) and total cholesterol (TC), termed TC-GNRI, in predicting pneumonia mortality in community-dwelling aged individuals. **Methods:** A longitudinal analysis of the Tsurugaya cohort in Japan, including 1124 participants aged 70 years or older, was used for tracking pneumonia-related deaths for an 11-year period. Total cholesterol thresholds were set at 171 mg/dL (males) and 192 mg/dL (females), classified into higher- and lower-groups. GNRI was divided into higher (≥100.64) and lower (<100.64) groups. A combined index (TC-GNRI) was then created by integrating these indices into three levels: high (both values at or above the thresholds), intermediate (one value above and one below), and low (both below). Cox proportional hazards models estimated hazard ratios (HRs) for pneumonia mortality, adjusting for age, gender, smoking, the Timed Up and Go test, %FVC, and tuberculosis, using high groups as references. **Results:** Kaplan–Meier curves showed that lower total cholesterol and lower GNRI were associated with higher pneumonia mortality than in the respective higher groups. The intermediate- and low-TC-GNRI groups had poorer survival rates than the high group. After adjustment, lower total cholesterol (HR = 3.03, 95% CI 1.41–6.52) and lower GNRI (HR = 2.69, 95% CI 1.21–5.99) were each associated with greater pneumonia mortality than the higher groups. The intermediate- (HR = 2.81, 95% CI 1.18–6.70) and low-TC-GNRI (HR = 6.17, 95% CI 2.15–17.74) groups showed greater pneumonia mortality than the high group. **Conclusions:** TC-GNRI may provide additional value in indicating pneumonia mortality risk than total cholesterol or GNRI alone. TC-GNRI may be a valuable tool for identifying older adults at particularly high risk of pneumonia mortality.

## 1. Introduction

The World Health Organization data consistently ranks pneumonia between 3rd and 5th among the top global causes of death [1]. Pneumonia and aspiration pneumonia together accounted for 8.4% of deaths, annually alternating between the third and fourth most prevalent cause of mortality in Japan, one of the world’s most aged societies [2]. Notably, pneumonia mortality is concentrated among the elderly, with individuals aged 70 and older accounting for 96% of these deaths [2].

Although malnutrition is a widely recognized predictor of pneumonia development and mortality [3,4,5], few longitudinal cohort investigations have examined the link between malnutrition and pneumonia mortality among community-dwelling populations of both genders. For instance, a previous male-only cohort study using body mass index (BMI) as a nutritional indicator found that higher BMI was related to a reduced mortality risk from respiratory disease [6]. Alternatively, the Geriatric Nutritional Risk Index (GNRI) is determined by body weight, plasma albumin, and height to objectively evaluate nutritional status in aged people. It was first developed to predict nutrition-related complications [7]. However, its predictive accuracy for pneumonia-related events remains debated; for example, a one-year follow-up study of patients in long-term nursing hospitals found that GNRI alone did not accurately predict pneumonia development in this frail population [8]. Few other prospective studies have applied this index for investigating pneumonia-related events. Consequently, it remains unclear whether GNRI can serve as a predictive index for pneumonia mortality among community-dwelling older adults.

In parallel with nutritional status, previous investigations reported that low total cholesterol is linked to increased mortality risk from infections, cancer, and non-cardiovascular diseases [9,10,11]. Regarding respiratory disease specifically, in a 7-year follow-up study of individuals aged over 40, lower total cholesterol was associated with increased mortality from respiratory failure [12]. Furthermore, in Asian chronic obstructive pulmonary disease (COPD) patients (mean age 69.1 years), high total cholesterol was associated with reduced risk of pneumonia development and improved overall mortality risk over an 8-year follow-up period [13]. Nevertheless, current evidence remains inconclusive. A 15-year follow-up of a multi-ethnic population (ages 15–79) in California found that low total cholesterol was associated with a higher risk of pneumonia hospitalization [14]. Conversely, a US community-based study (ages 45–64) found no association with pneumonia hospitalization over a median follow-up of >20 years [15]. Although total cholesterol may potentially be associated with respiratory diseases, particularly pneumonia mortality, the results remain inconclusive. Therefore, in addition to simply accumulating further cohort studies, we speculated that combining total cholesterol with another indicator might create a more precise predictive indicator.

Indeed, previous studies have shown that combining GNRI with another marker improved its predictive value. For example, in hemodialysis patients, combining GNRI with the modified creatinine index improved its mortality prediction [16]. Similarly, in heart diseases, combining GNRI with hemoglobin better predicted in-hospital mortality or adverse cardiovascular events than hemoglobin or GNRI alone [17,18]. In cancer surgery, combining GNRI with hemoglobin better predicted survival after pancreaticoduodenectomy surgery than hemoglobin or GNRI alone [19]. In surgical patients with gastric or colorectal cancer, GNRI combined with calf circumference predicted overall survival rates more precisely than either GNRI or calf circumference alone [20]. Thus, combining GNRI with another indicator may enhance mortality risk prediction compared with using either indicator alone.

Overall, while numerous studies have reported an inverse association between total cholesterol and pneumonia-related mortality, several conflicting findings have also been documented. Similarly, although the association between malnutrition and increased pneumonia mortality is well recognized, it is not consistently observed across prior reports. We hypothesized that these discrepancies in the previous literature may arise from the limitations of using either marker in isolation. Therefore, we speculated that integrating these two indicators would enable a more comprehensive identification of high-risk elderly populations.

Malnutrition contributes to the decline in respiratory muscle strength, which subsequently weakens coughing and elevates the risk of pneumonia mortality [3,4,21,22]. Concurrently, low total cholesterol levels may impair the immune responses, potentially compromising the host’s defense mechanisms [23]. Malnutrition also impairs immune responses [3,4]. These data suggest the potential of integrating nutritional status and lipid profiles for a comprehensive assessment of pneumonia mortality risk. Given that these indicators represent distinct physiological domains with minor overlaps, we speculated that combining these two different parameters would be highly effective for identifying older adults at high risk of pneumonia mortality.

Currently, no studies have yet combined GNRI with another indicator to predict pneumonia mortality among elderly people. To explore the potential complementary value of total cholesterol (TC) and GNRI, we first examined their correlation within our cohort. Subsequently, we integrated these measures into a new composite parameter, TC-GNRI, with the primary aim of assessing its association with pneumonia mortality.

## 2. Materials and Methods

### 2.1. Participants

The Tsurugaya cohort study was initiated in 2002 and recruited community residents aged 70 years and above residing in the Tsurugaya district of Sendai, Japan [21]. The initial baseline parameters were gathered utilizing a standardized Comprehensive Geriatric Assessment. Regarding ethical considerations, the Ethics Committee at Tohoku University Graduate School of Medicine approved this study, which was conducted in full accordance with the principles of the Declaration of Helsinki (approval number 2002-040, approved on 20 May 2002). Each participant gave their written informed consent.

### 2.2. Gathering of Clinical and Laboratory Data

Initially, non-fasting venous blood was collected and processed by a certified laboratory for serum lipids and albumin, including total cholesterol. To determine the optimal thresholds, gender-specific total cholesterol thresholds were set at 171 mg/dL (males) and 192 mg/dL (females) using X-tile (v3.6.1, Yale University) with pneumonia-related mortality as the endpoint. In addition to blood samples, questionnaires recorded demographics and medical history (myocardial infarction, stroke, tuberculosis, pneumonia, asthma, hypertension, and diabetes), marital status, smoking (current/former/never), and statin use. Furthermore, pulmonary function, including % predicted value forced vital capacity (%FVC) and forced expiratory volume in 1 s (FEV1), was evaluated three times using a spirometer (OST-80A; Chest Co., Tokyo, Japan), and the greatest value was chosen. %FVC was calculated from height, age, and gender. Moreover, in the Timed Up and Go test, we recorded the time it took subjects to stand from a chair, walk 3 m, turn around, return, and sit down again. Finally, cognitive status and depressive symptoms were quantified through the Japanese translation of the Mini-Mental State Examination and the 30-item geriatric depression scale (GDS-30), respectively [22].

### 2.3. Calculation of GNRI

We assessed body mass index (BMI) by weight (kg) and height (m). Subsequently, we evaluated nutritional status with the GNRI, defined as 14.89 × serum albumin (g/dL) + 41.7 × (actual body weight/ideal body weight). The ideal body weight was calculated differently for each gender, height (cm) − 100 − [(height − 150)/4] for males, and height (cm) − 100 − [(height − 150)/2.5] for females. Similar to the cholesterol analysis, the GNRI threshold was set at 100.64 using X-tile; accordingly, values were classified as <100.64 or ≥100.64.

### 2.4. TC-GNRI Scoring System

We defined a three-level stratification for TC-GNRI based on thresholds for total cholesterol and GNRI: a score of 2 (both total cholesterol and GNRI at or above their thresholds); a score of 1 (one above and one below); and a score of 0 (both below). This approach integrates lipid and nutritional information to provide a comprehensive framework for risk stratification. Additionally, in a sensitivity analysis, we replicated the main predictive models by excluding individuals who succumbed within the first 2 years of follow-up.

### 2.5. Follow-Up Study

The International Classification of Diseases, 10th Revision, (ICD-10) standards classified mortality causes. In this study, the primary outcome was pneumonia mortality labeled as J12–18, J20, and J69. Death-related information was collected from the Sendai City Government. Hospital documentation or the information transmitted to the Japan Arteriosclerosis Longitudinal Study Coordinating Center was reviewed. The Tsurugaya Project was part of the Japan Arteriosclerosis Longitudinal Study, which gathered 21 Japanese cohorts. Overall, pneumonia deaths were tracked until 1 July 2012 [22].

### 2.6. Statistical Analysis

Statistical analyses were executed by IBM SPSS Statistics (version 24.0, IBM Corp., Armonk, NY, USA). First, the optimal cutoff values for total cholesterol and the GNRI were identified by X-tile software (v3.6.1, Yale University) with time to pneumonia death as the primary endpoint. Next, differences in baseline clinical factors across the resulting TC-GNRI groups were assessed utilizing a one-way analysis of variance (ANOVA). For survival analysis, survival curves stratified by total cholesterol, GNRI, and TC-GNRI scores were generated using the Kaplan–Meier method and compared using the log-rank test. To identify factors independently associated with pneumonia mortality, we performed Cox proportional hazards regression to obtain Hazard Ratios (HR) and 95% Confidence Intervals (CI). Two models were used to evaluate the link between total cholesterol, GNRI, TC-GNRI, and pneumonia mortality: Model 1 (crude model), Model 2 (adjustment for gender, age, smoking, Timed Up and Go test, %FVC, and tuberculosis). In a sensitivity analysis, we repeated the Cox proportional hazards models after excluding participants who died within the first 2 years of follow-up, in order to reduce potential reverse causality. A bootstrapping analysis with 1000 resamples estimated hazard ratios (95% CI) of the TC-GNRI groups using the bias-corrected and accelerated method. The concordance statistic (C-statistic) was used to assess the model’s ability to discriminate between participants with and without the events. A C-statistic of 0.5 indicates no discrimination, while 1.0 indicates perfect discrimination. To evaluate the potential for interaction between GNRI and total cholesterol, we included a multiplicative interaction term in the Cox proportional hazards models. A *p*-value of 0.05 was adopted for assessing statistical significance.

## 3. Results

**Figure 1:**
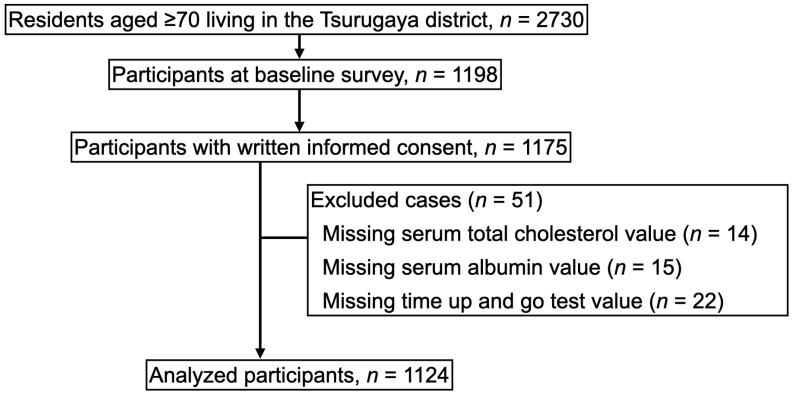
Study flow chart. In 2002, of the 2730 residents invited, 1175 provided informed consent. We excluded 51 participants due to missing baseline data (total cholesterol, n = 14; albumin, n = 15; Timed Up and Go Test, n = 22). The final analysis included 1124 community-dwelling older adults.

In 2002, the Tsurugaya Project invited all 2730 residents aged 70 years and older residing in the Tsurugaya region to participate. The baseline survey included 1198 individuals, with 1175 participants providing informed consent. Figure 1 presents the study flow. From this initial group, we excluded 51 participants due to missing data: 14 lacked total cholesterol, 15 lacked serum albumin, and 22 lacked Timed Up and Go results. The exclusion of participants was based solely on the availability of complete data. Consequently, the final analysis incorporated 1124 elderly individuals.

**Figure 2:**
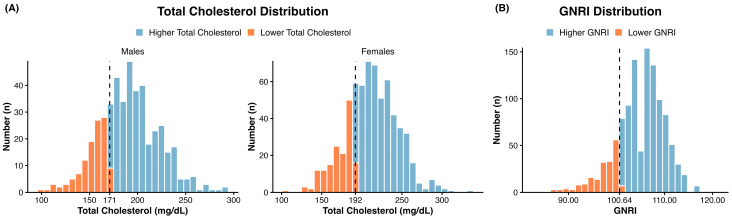
Optimal total cholesterol and GNRI thresholds. (**A**) Total cholesterol distribution and its thresholds in males (left panel, 171 mg/dL) and females (right panel, 192 mg/dL). (**B**) GNRI distribution and its cutoff value (100.64).

Participants were categorized into 2 groups, higher and lower, based on their total cholesterol and GNRI values, respectively. The thresholds were calculated using X-tile with pneumonia-related death as the endpoint. The total cholesterol thresholds differed by gender, males (171 mg/dL) and females (192 mg/dL, Figure 2A). Since GNRI was calculated differentially by gender, the threshold of 100.64 was applied to both genders (Figure 2B). The standard cutoff value for malnutrition using the GNRI is generally established at 98. In the current study, the optimal cutoff value determined by X-tile software was 100.6, which is closely aligned with the clinical standard. We interpreted this minimal discrepancy supports the validity and clinical relevance of using X-tile for our data stratification. Thus, we observed a convergence between the statistically optimized value and the practical clinical threshold for the GNRI cut-off. Furthermore, the GNRI threshold used in this study was not intended to redefine clinical nutritional categories but rather to optimize risk stratification for pneumonia mortality among older adults. Regarding total cholesterol, while the upper limit of the normal range is well-defined and clinically significant, a clear lower limit remains controversial. Therefore, we utilized X-tile software to determine an objective cutoff value tailored to our study population. Thus, for total cholesterol, we used a data-driven approach in X-tile software to determine the threshold.

Before combining these indices, multicollinearity was evaluated using variance inflation factors. The variance inflation factor for GNRI and total cholesterol was 1.11, indicating minimal multicollinearity between these variables. GNRI and total cholesterol showed a modest correlation, with a correlation coefficient of 0.315, suggesting these parameters can capture different aspects in pneumonia mortality prediction. Thus, we combined total cholesterol and GNRI and named TC-GNRI to predict pneumonia mortality. Referring to the previously developed combination of hemoglobin and GNRI, we classified TC-GNRI into three groups: high, intermediate, and low [19]. Accordingly, we defined both total cholesterol and GNRI values at or above their thresholds as a score of 2 (a high group, n = 750), one above and one below as a score of 1 (an intermediate group, n = 289), and both below as a score of 0 (a low group, n = 85). When stratified by this three-level score, a low TC-GNRI score was signifiantly associated with advanced age, current smokers, worse (slower) Timed Up and Go test, and an increased number of a history of tuberculosis. Baseline characteristics of the groups divided by TC-GNRI scores are summarized in Table 1.

**Figure 3:**
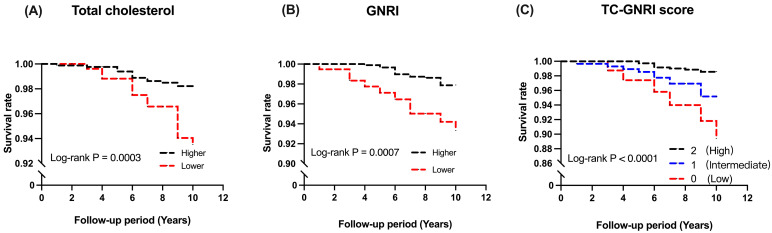
Kaplan–Meier survival curves for pneumonia mortality: (**A**) Total cholesterol: higher vs. lower (thresholds: 171 mg/dL for males, 192 mg/dL for females). (**B**) GNRI: higher vs. lower (threshold: 100.6). (**C**) TC-GNRI: high group (score 2, both total cholesterol and GNRI at or above their thresholds), intermediate group (score 1, one above and one below), and low group (score 0, both below).

Over the following period, 28 pneumonia deaths were observed. Kaplan–Meier survival curves showed better pneumonia survival in the higher total cholesterol (*p* = 0.0003; Figure 3A) and GNRI (*p* = 0.007; Figure 3B) groups than in their respective lower groups. Notably, the pneumonia survival rate showed a significant stepwise decline across the 3 TC-GNRI groups (high > intermediate > low, *p* = 0.0001, Figure 3C). In terms of absolute numbers, the survival rates were 98.6% (high group), 95.2% (intermediate group), and 89.4% (low group).

Among 28 pneumonia deaths, 14 occurred in participants with higher total cholesterol (n = 856) and 14 in those with lower total cholesterol (n = 268), whereas 18 occurred in participants with higher GNRI (n = 933) and 10 in those with lower GNRI (n = 191). Table 2 divided total cholesterol and GNRI values into higher and lower groups and showed their relationship with pneumonia mortality. Each used the higher group as a reference. In the crude model, the lower total cholesterol group had a greater pneumonia mortality risk than the higher group. Similarly, the lower GNRI group had a greater risk than the higher group. Regarding the selection of adjustment variables, we utilized a dual approach. First, we prioritized variables identified in the previous literature as risk factors for pneumonia mortality, such as advanced age, male sex, smoking, and low %FVC [22]. Second, we included factors that exhibited statistically significant differences across the TC-GNRI groups in Table 1 (e.g., TUG and history of tuberculosis). Among the factors with significant differences identified in Table 1, GNRI is calculated from albumin and body mass index. Therefore, we selected gender, age, smoking, the Timed Up and Go test, %FVC, and tuberculosis as adjustment variables. After adjusting for these covariates, lower total cholesterol was associated with a greater risk of pneumonia mortality than the higher values (Model 2: HR = 3.03; 95% CI, 1.41–6.52). Similarly, the lower GNRI was associated with a greater risk than the higher values (Model 2: HR = 2.69, 95% CI: 1.21–5.99).

Table 3 showed the relationship between TC-GNRI divided into three groups and pneumonia mortality, using the high group (score = 2, n = 750) as a reference. Hazard ratios (95% CIs) in the crude model were 3.40 (1.47–7.86) for the intermediate group (score = 1, n = 289) and 7.29 (2.64–20.08) for the low group (score = 0, n = 85). In the adjusted model, the intermediate group had a greater risk of pneumonia mortality than the high group (Model 2: HR 2.81, 95% CI: 1.18–6.70). The low group (score = 0, n = 85) also showed a greater mortality risk (Model 2: HR 6.17, 95% CI: 2.15–17.74), with a *p*-for-trend (*p* < 0.001) across the 3 groups. To ensure the validity of these Cox models, we assessed the proportional hazards assumption for the Cox models using log-minus-log survival plots. The proportional hazards assumption was evaluated for the main exposure variables (total cholesterol, GNRI, and TC-GNRI), and the results satisfied the criteria for the Cox regression analysis.

To further validate the robustness of our results, we considered the possibility that poor health conditions at the time of measurement might have worsened the TC-GNRI score (reverse causality). Accordingly, we excluded cases that died within two years after measurement and examined the link between TC-GNRI and pneumonia mortality risk as a sensitivity analysis. Twenty-seven individuals died from pneumonia, but there was no essential change in the results. Using the high group (score = 2, n = 743) as a reference, in the adjusted model, the HR for the intermediate group (score = 1, n = 279) was 2.75 (95% CI: 1.14–6.60), and the HR for the low group (score = 0, n = 82) was 7.69 (95% CI: 2.72–21.75), with a *p*-for-trend (*p* < 0.001) across the 3 groups (Supplementary Table S1).

We next assessed the robustness of our findings, as the relatively small number of events could potentially lead to overfitting given the six adjustment factors. A bootstrap analysis with 1000 resamples was performed, and the results remained rather stable with the primary analysis (Supplementary Table S2). After adjustment, the intermediate group exhibited a significantly higher risk of pneumonia mortality compared to the high group (Model 2: HR 2.67; 95% CI: 1.10–6.51). Similarly, the low group showed an increased mortality risk (Model 2: HR 4.82; 95% CI: 1.60–14.52), with a significant trend across groups (*p*-for-trend < 0.02).

Furthermore, we evaluated model fit statistics to support the improved identification of high-risk participants (Supplementary Table S3). The C-statistic increased from 0.68 in Model 1 to 0.82 in Model 2, suggesting better discrimination. Finally, we tested for an interaction between GNRI and total cholesterol; however, no significant interaction was observed (*p* = 0.71).

## 4. Discussion

Overall, our survival rate analysis showed a stepwise association between TC-GNRI scores and pneumonia mortality, with the low TC-GNRI group having the poorest survival outcomes. Compared with the high TC-GNRI group, the adjusted pneumonia mortality risk was approximately 2.8 times higher in the intermediate group and 6.5 times higher in the low group. These associations suggested that worsening in TC-GNRI scores was linked to a greater risk of pneumonia mortality. Furthermore, our data suggest that TC-GNRI may better identify individuals at greater risk of pneumonia mortality compared to total cholesterol or GNRI alone.

While the link between malnutrition and the risk of pneumonia onset or mortality has been established in previous studies, our research suggested this relationship in a longitudinal cohort of both genders, a finding rarely reported before. Furthermore, we suggested the effectiveness of GNRI as an indicator of malnutrition. In addition to nutritional status, low total cholesterol was also suggested to be associated with pneumonia mortality among community-dwelling older people.

Regarding the underlying biology, the immune system might be one of the mechanisms by which low total cholesterol was related to pneumonia mortality. Indeed, recent research reported a part of the largely unclear association between cholesterol and immune networks. The research showed that cholesterol contributed to protection from cell death in CD4-positive T cells [23], which occupy central roles in immune networks [24]. In addition, cholesterol may play essential roles in the host immune system, such as directly binding to the bacteria or promoting phagocytosis of macrophages [15,25]. However, due to inconsistent results in previous studies regarding the relationship between total cholesterol and pneumonia mortality, we combined the nutritional indicator GNRI with total cholesterol and created TC-GNRI. Notably, the correlation between GNRI and total cholesterol was relatively weak (r = 0.315), suggesting that they provide complementary information for predicting pneumonia mortality. This relatively low correlation suggests that combining these complementary parameters into TC-GNRI may provide a more comprehensive prediction of pneumonia mortality risk. In addition, the variance inflation factor of 1.11 for GNRI and total cholesterol suggested their minimal multicollinearity. While a higher GNRI may reflect greater muscle strength for effective coughing, higher total cholesterol may reflect robust immune function. Therefore, combining these two statistically distinct factors is considered meaningful for identifying elderly individuals at high risk of pneumonia mortality.

When participants were classified into 3 categories according to TC-GNRI scores, the survival curves for pneumonia mortality showed clear separation among the groups. Worsening in TC-GNRI was associated with lower survival rates. Hazard ratios showed similar trends, with a statistically significant *p*-value for the trend between the 3 groups. These results suggested that TC-GNRI may provide additional insight into pneumonia mortality risk beyond total cholesterol or GNRI alone.

Interestingly, although lower total cholesterol was associated with a higher pneumonia mortality risk, the use of statins did not appear to be linked to an increased risk. In fact, prior studies suggested that statin therapy might be associated with reduced mortality in patients with pneumonia, COPD exacerbations, and interstitial pneumonia [26,27,28,29,30]. These benefits may help mitigate the risk associated with low total cholesterol by taking statins.

From a clinical perspective, TC-GNRI is composed of routinely measured clinical parameters, including body weight, height, serum albumin, and total cholesterol, which are commonly available in standard clinical examinations. This simplicity may facilitate its application as a risk stratification tool among older adults. Thus, TC-GNRI may provide complementary information to support early identification of older individuals at higher risk of pneumonia mortality. Consequently, the TC-GNRI categories are expected to function as a practical tool in outpatient, home-care, and hospital settings, enabling clinicians to identify high-risk older individuals and consider preventive or interventional strategies.

Despite the above findings, our study cohort was limited to Japanese older adults in a single community, which may restrict the generalizability of the results to other ethnic groups with different dietary patterns and healthcare infrastructures. For instance, differences in healthcare access across countries could affect mortality outcomes. Furthermore, it has been reported that East Asian populations, such as Japanese, tend to have lower respiratory muscle strength and ineffective coughing compared to other ethnic groups, including older adults [3,4]. Therefore, future multi-ethnic and international studies are required to confirm the universal applicability of combining GNRI and total cholesterol as a risk assessment tool.

Another potential limitation of this study is the relatively small number of events (n = 28), which may have led to overfitting given that six adjustment factors were included in the multivariable model. However, to address this concern and ensure the stability of our findings, we performed a bootstrapping analysis with 1000 resamples. The results remained consistent with the original model, suggesting that the risk of overfitting was limited and that our estimates were relatively robust.

Additionally, we lacked information on lipid-lowering medications other than statins, such as fibrates. Furthermore, we lacked data on other drugs that may affect nutritional or immune status, such as anti-rheumatic drugs. However, we identified oral steroid use as a relevant parameter for further analysis. Our analysis revealed no significant differences in steroid utilization among the three TC-GNRI groups (*p* = 0.23): high (n = 32, 4.3%), intermediate (n = 8, 2.8%), and low (n = 5, 5.9%). Nevertheless, this finding alone cannot fully mitigate the risk of bias arising from other undocumented medications. The potential for ‘residual confounding’ due to missing medication data remains a constraint, and our findings must therefore be interpreted with caution.

Some participants might already be frail or had undiagnosed illness at baseline and died soon after enrollment. In such cases, a low GNRI or low total cholesterol might likely reflect an underlying illness. To avoid this possibility, we re-analyzed the main result after removing deaths in the initial two years of the following period. While the results remained consistent, it is still possible that low GNRI and total cholesterol levels serve as proxy markers for underlying chronic illness or frailty. Furthermore, regarding risk factors for pneumonia-related mortality, we acknowledge that swallowing function—a critical variable—was not assessed in our cohort. Consequently, we were unable to include it as a covariate in our multivariate models, which may result in residual confounding.

To date, there are limited reports on risk scores for identifying community-dwelling elderly individuals at high risk of pneumonia mortality. Known risk factors include advanced age, male sex, cognitive decline, comorbidities (respiratory and cardiovascular diseases, diabetes), smoking, swallowing dysfunction, and low %FVC [22]. In this study, we assessed whether these factors, excluding swallowing function due to data unavailability, showed significant differences in distribution across the three TC-GNRI groups (high, intermediate, and low). Factors with confirmed differences were subsequently adjusted for in our analysis. While it is challenging to account for all variables, a comprehensive comparison of these risk factors with TC-GNRI will be a key objective for future research to further validate TC-GNRI’s utility.

Our research includes several limitations as shown above. In short, the populations were predominantly Japanese/East Asian, with a relatively homogeneous ethnic composition. Second, the pneumonia mortality count was insufficient for various adjustments. The relatively small number of pneumonia-related deaths (n = 28) might have limited the statistical power to detect significant differences and may have affected the stability of the hazard ratio estimates. It is widely recognized that a minimum of 10 events per variable is recommended to ensure robustness and stability in multivariable models. However, our events per variable were approximately 4.67, given the six adjusted variables. This relatively low number of events per variable suggests that the risk of overfitting cannot be entirely ruled out. Third, analyzing the effects of cholesterol-lowering medications other than statins, such as ezetimibe, was impossible. Fourth, regarding risk factors for pneumonia-related mortality, we acknowledge that swallowing function—a critical variable—was not assessed in our cohort. Consequently, we were unable to include it as a covariate in our multivariate models.

## 5. Conclusions

In summary, among community-dwelling elderly individuals, lower total cholesterol and lower GNRI values were each associated with increased pneumonia mortality, and their combination, the TC-GNRI, appeared to provide additional insight into pneumonia mortality risk compared with either measure alone. While a high total cholesterol induces a greater mortality risk in cardiovascular diseases, our study suggests that excessively low total cholesterol may be associated with an increased risk of pneumonia mortality. Importantly, total cholesterol, albumin, height, and weight are routinely measured in daily clinical practice; therefore, calculating TC-GNRI does not require any additional examinations. In conclusion, as aging progresses and pneumonia deaths continue to increase globally, the easily calculated TC-GNRI may have a role to play as a simple tool for assessing pneumonia mortality risk.

## Figures and Tables

**Table 1:** Baseline characteristics of participants stratified by TC-GNRI score.

	TC-GNRI Score				
Characteristics	Overall	2 (High)	1 (Intermediate)	0 (Low)	*p*-Value
Number	1124	750	289	85	
Gender					0.389
Female, n (%)	657 (58.5%)	449 (59.8%)	160 (55.4%)	48 (56.5%)	-
Men, n (%)	467 (41.5%)	301 (40.1%)	129 (44.6%)	37 (43.5%)	-
Age, mean (SD)	75.7 (4.8)	75.2 (4.6)	76.7 (5.2)	76.8 (4.8)	<0.001
Body mass index (kg/m^2^), mean (SD)	23.9 (3.4)	24.6 (3.0)	23.1 (3.6)	20.3 (3.2)	<0.001
Albumin (g/dL), mean (SD)	4.3 (0.3)	4.4 (0.2)	4.2 (0.3)	3.9 (0.3)	<0.001
Geriatric Nutritional Risk Index	104.9 (5.0)	106.8 (3.5)	102.7 (5.1)	96.2 (3.9)	<0.001
Total cholesterol (mg/dL), mean (SD)	203.8 (33.4)	217.7 (26.4)	180.7 (28.6)	159.9 (19.8)	<0.001
Mini-Mental State Examination	26.5 (9.7)	26.9 (8.5)	25.6 (13.2)	26.5 (3.5)	0.171
Taking statins, n (%)	189 (16.8%)	131 (17.5%)	51 (17.7%)	7 (8.2%)	0.089
Timed Up and Go test	9.9 (3.5)	9.8 (3.4)	10.0 (3.4)	10.9 (4.3)	0.033
Current Smoker, n (%)	141 (12.5%)	84 (11.2%)	38 (13.2%)	19 (22.4%)	0.012
Marital status, n (%)	677 (60.2%)	459 (61.2%)	164 (56.8%)	54 (63.5%)	0.342
Depressive symptoms, mean (SD)	8.4 (10.7)	8.6 (9.7)	8.3 (10.7)	7.3 (17.5)	0.566
FVC (L), mean (SD)	1.5 (10.9)	1.3 (11.2)	1.8 (11.9)	2.3 (0.8)	0.611
% FVC, mean (SD)	94.3 (24.0)	94.8 (23.4)	94.8 (25.9)	88.2 (21.8)	0.051
FEV_1_/FVC % (SD)	72.9 (14.6)	72.7 (13.3)	73.1 (17.9)	74.3 (13.1)	0.613
Medical History					
Myocardial infarction, n (%)	124 (11.0%)	82 (10.9%)	33 (11.4%)	9 (10.6%)	0.966
Stroke, n (%)	60 (5.3%)	39 (5.2%)	18 (6.2%)	3 (3.5%)	0.597
Tuberculosis, n (%)	153 (13.6%)	87 (11.6%)	49 (16.9%)	17 (20%)	0.016
Pneumonia, n (%)	105 (9.3%)	62 (8.3%)	30 (10.4%)	13 (15.3%)	0.084
Asthma, n (%)	74 (6.6%)	48 (6.4%)	19 (6.6%)	7 (8.2%)	0.811
Hypertension, n (%)	431 (38.3%)	299 (39.9%)	106 (36.7%)	26 (30.6%)	0.198
Diabetes, n (%)	155 (13.8%)	104(13.9%)	42 (14.5%)	9 (10.6%)	0.647

Groups are defined as High (score 2), Intermediate (score 1), and Low (score 0). Data are presented as mean (standard deviation [SD]) or n (%). forced vital capacity (FVC); % predicted value FVC (% FVC); forced expiratory volume in 1 s (FEV1).

**Table 2:** Association of total cholesterol and GNRI with pneumonia mortality *.

	**Higher Total Cholesterol**	**Lower Cholesterol**	***p*** **-Value**
	**(n = 856)**	**(n = 268)**	
Pneumonia deaths (n)	14	14	
Model 1	1.00 (Reference)	3.54 (1.69–7.44)	0.001
Model 2	1.00 (Reference)	3.03 (1.41–6.52)	0.005
	**Higher GNRI**	**Lower GNRI**	
	**(n = 933)**	**(n = 191)**	
Pneumonia deaths (n)	18	10	
Model 1	1.00 (Reference)	3.38 (1.56–7.33)	0.002
Model 2	1.00 (Reference)	2.69 (1.21–5.99)	0.016

* Values are HR (95% CI). Model 1: Crude; Model 2: Adjusted for gender, age, smoking, Timed Up and Go test, %FVC, and tuberculosis.

**Table 3:** Relationship between TC-GNRI scores and pneumonia mortality.

	TC-GNRI Score			
	2 (High) (n = 750)	1 (Intermediate) (n = 289)	0 (Low) (n = 85)	*p*-Trend
Pneumonia deaths (n)	10	12	6	
Model 1	1.00 (Reference)	3.40 (1.47–7.86) *	7.29 (2.64–20.08) ^§^	<0.001
Model 2	1.00 (Reference)	2.81 (1.18–6.70) *	6.17 (2.15–17.74) ^§^	<0.001

Values are HR (95% CI). Model 1: Crude; Model 2: Adjusted for gender, age, smoking, Timed Up and Go test, %FVC, and tuberculosis. * *p* < 0.02, ^§^ *p* < 0.001 vs. High group.

## Data Availability

The data presented in this study are available on request from the corresponding author due to ethical restrictions.

## Supplementary Materials

The following supporting information can be downloaded at: https://www.mdpi.com/article/10.3390/nu18030465/s1. Supplemental Table S1: Association between TC-GNRI score and pneumonia mortality excluding deaths within the first 2 follow-up years. Supplemental Table S2: Bootstrap validation (1000 resamples) of the multivariable Cox model for pneumonia mortality. Supplement Table S3: Model fit and discrimination statistics for pneumonia mortality.

## Abbreviations

hazard ratios (HRs); confidence intervals (CIs); Geriatric Nutritional Risk Index (GNRI); total cholesterol (TC); total cholesterol combined with Geriatric Nutritional Risk Index (TC-GNRI); Chronic obstructive pulmonary disease (COPD); body mass index (BMI); Geriatric Depression Scale (GDS); Mini-Mental State Examination (MMSE); The International Statistical Classification of Diseases and Related Health Problems 10th Revision (ICD-10); standard deviation (SD); forced vital capacity (FVC); % predicted value FVC (% FVC); forced expiratory volume in 1 s (FEV1).
