# Supplementary material for: Combining Geriatric Nutritional Risk Index with Total Cholesterol to Predict Pneumonia Mortality Risks in a Cohort of General Older Adults
Source: Nutrients. 2026 Jan 30;18(3):465. doi: 10.3390/nu18030465 (PMC12899913; doi:10.3390/nu18030465)
Supplement: Supplementary file 1 [file nutrients-18-00465-s001.zip › nutrients-4086503-supplementary.pdf]

Supplemental Table S1. Association between TC-GNRI score and pneumonia mortality excluding deaths within the first 2 follow-up years.

	TC-GNRI score<br>2<br>(High)<br>(n = 743)	 1<br>(Intermediate)<br>(n = 279)	 0<br>(Low)<br>(n = 82)	*p*-for trend
Pneumonia deaths (n)	10	11	6	
Model 1	1.00 (Reference)	3.12 (2.68-7.35)*	7.38 (2.68-20.34)§	< 0.001
Model 2	1.00 (Reference)	2.75 (1.14-6.60)*	7.69 (2.72-21.75)§	< 0.001

Values are HR (95% CI). Model 1: Crude; Model 2: Adjusted for gender, age, smoking, Timed Up and Go test, %FVC, and tuberculosis. *$p < 0.02$, §$p < 0.001$ vs. High group.

Supplemental Table S2. Bootstrap validation (1,000 resamples) of the multivariable Cox model for pneumonia mortality.

	TC-GNRI score<br>2<br>(High)<br>(n = 743)	 1<br>(Intermediate)<br>(n = 279)	 0<br>(Low)<br>(n = 82)	*p*-for trend
Pneumonia deaths (n)	10	11	6	
Model 1	1.00 (Reference)	3.21 (1.37-7.50)*	5.62 (1.99-15.88)§	0.002
Model 2	1.00 (Reference)	2.67 (1.10-6.51)*	4.82 (1.60-14.52)§	0.012

Values are HR (95% CI). Model 1: Crude; Model 2: Adjusted for gender, age, smoking, Timed Up and Go test, %FVC, and tuberculosis. *$p < 0.02$, §$p < 0.001$ vs. High group.

Supplemental Table S3. Model fit and discrimination statistics for pneumonia mortality.

	TC-GNRI score			C-statistic
	2 (High) (n = 750)	1 (Intermediate) (n = 289)	0 (Low) (n = 85)	
Pneumonia deaths (n)	10	12	6	
Model 1	1.00 (Reference)	3.40 (1.47-7.86)*	7.29 (2.64-20.08)§	0.68
Model 2	1.00 (Reference)	2.81 (1.18-6.70)*	6.17 (2.15-17.74)§	0.82

Values are HR (95% CI). Model 1: Crude; Model 2: Adjusted for gender, age, smoking, Timed Up and Go test, %FVC, and tuberculosis. *$p < 0.02$, §$p < 0.001$ vs. High group.
